# Wzy 3D structural models correlate with inter-repeat unit glycosidic bond configuration in pneumococcal capsule polysaccharides

**DOI:** 10.1128/spectrum.00328-25

**Published:** 2025-09-03

**Authors:** Feroze A. Ganaie, Melissa B. Oliver, Jamil S. Saad, David G. Glanville, Andrew T. Ulijasz, Moon H. Nahm

**Affiliations:** 1 Division of Pulmonary, Allergy and Critical Care, Department of Medicine, The University of Alabama at Birmingham 654677 , Birmingham, Alabama, USA; 2 Department of Microbiology, The University of Alabama at Birmingham 318277, Birmingham, Alabama, USA; University of Heidelberg, Heidelberg, Germany

**Keywords:** bacterial surface glycan polymerase, Wzy, AlphaFold, *Streptococcus pneumoniae*, capsule

## Abstract

Bacterial surface glycan polymerases, such as Wzy, are integral membrane-bound glycosyltransferases that synthesize various surface-bound glycopolymers by linking repeat units via α-glycosidic or β-glycosidic bonds. Despite its central role in the widely employed “Wzy/Wzx-dependent pathway” for glycan synthesis, Wzy remains poorly understood, largely due to its high sequence variability. Using *Streptococcus pneumoniae* (pneumococcus) capsules as a model, we leveraged AlphaFold and Orientation of Proteins in Membranes computational tools to predict 3D molecular architectures of pneumococcal Wzys and elucidate their correlation with glycosidic linkage stereochemistry. Predicted structures revealed two distinct Wzy types with high confidence (average predicted local distance difference test score ≥85; 95% confidence interval: 85.86–86.01): type-A and type-B, distinguished by the cavity orientation and partly by differing C-terminus topology. Predictions showed that type-A Wzy models featured a cavity extending toward the cytoplasm and were predominantly associated with α-glycosidic bonds. In contrast, type-B Wzy models displayed a cavity oriented toward the extracellular interface and primarily catalyze β-glycosidic bonds. To facilitate sequence-based classification of type-A and type-B Wzys, we identified two location-specific conserved motifs, GN1 and GN2, predominantly found in type-B Wzys and strongly associated with β-configuration. Our findings offer critical insights into the Wzy structure-function relationship, highlighting its role in the stereochemical control of glycan polymerization. Although focused on pneumococci, these findings may extend to other bacteria. Further studies across species are needed to confirm and expand this structural-glycosidic bond relationship with experimental evidence.

**IMPORTANCE:**

Bacterial surface glycan polymerases, like Wzy, are key players in the synthesis of complex sugar-based polymers that form protective layers on the bacterial surfaces. However, the structure-function relationship of Wzy remains poorly defined due to its high sequence variability and membrane-bound nature. This study provides the first structural framework for classifying pneumococcal Wzy enzymes using AlphaFold-predicted 3D models in combination with Orientation of Proteins in Membranes computational tool, revealing two distinct structural types (type-A and type-B) that strongly correlate with glycosidic linkage stereochemistry. The identification of conserved sequence motifs further enables sequence-based classification, offering a valuable tool for studying Wzy across bacterial species. These findings not only advance our fundamental understanding of glycan polymerization but also have broader implications for bacterial pathogenesis and vaccine design. By establishing a direct link between Wzy structure and glycosidic bond formation, these findings provide a blueprint for exploring Wzy-dependent glycopolymer synthesis in other bacterial species, paving the way for targeted interventions in bacterial surface glycan biosynthesis.

## INTRODUCTION

Bacterial cell surfaces are decorated with many complex glycopolymers like capsule polysaccharide (CPS), lipopolysaccharide (LPS), exopolysaccharide, enterobacterial common antigen, and peptidoglycan. As the primary interface between bacteria and their environment (1), these glycopolymers play vital roles in a wide variety of biological processes, like attachment–colonization, persistence, and motility, which can influence infection, virulence, and interaction with host defense systems in a susceptible host (1, 2). These surface glycopolymers are generally polymerized by integral membrane-bound glycosyltransferases (GTs) (3–6) of the GT-C fold family that use diphospholipid-activated sugars (7, 8).

One such GT used for the polymerization of many CPS and LPS O-antigens is known as “Wzy,” a bacterial surface glycan polymerase (3, 8–10). Wzy is the main orchestrator of the “Wzy/Wzx-dependent pathway,” a widely used glycan synthesis mechanism employed by a large proportion of bacterial species (3, 11–13). This pathway involves the cytoplasmic synthesis of oligosaccharide repeat units (RUs), which are assembled on undecaprenyl phosphate (Und-P) lipid anchors. Once synthesized, the RUs are transported across the bacterial membrane by the Wzx flippase. At the external surface, Wzy polymerizes the pre-assembled RUs by linking them via either α-glycosidic or β-glycosidic linkages into long glycan chains.

At present, little is known about the Wzy proteins and their mechanism of action, especially in gram-positive bacteria (3, 10, 14–16). This limitation is primarily due to the challenge of expressing Wzy heterologously and the large sequence diversity across Wzy family members, which works with diverse donor and acceptor molecules to presumably accommodate highly variable polysaccharide structural makeup (10, 17–19). Furthermore, functional studies are hindered by the difficulty in making polyisoprenyl pyrophosphate-linked oligosaccharides required for *in vitro* assays. Interestingly, integral membrane-bound GTs associated with the GT-C fold, such as Wzy proteins, were traditionally thought to form only β-configurations until recently (7, 8), and there is virtually no knowledge about Wzys forming stereochemically different α-configuration. As members of the GT-C fold family (7, 8), Wzy enzymes may either retain or invert the anomeric configuration of the donor substrate. Inverting integral membrane-bound GTs likely employ an S_N_2-like displacement mechanism, whereas retaining integral membrane-bound GTs may utilize a less defined S_N_i-like mechanism (7, 8).

Predicting the stereochemistry of glycosidic bonds formed by Wzy based on sequence or transmembrane topology prediction algorithms has been historically unreliable (3). However, new advances in computational tools, such as “AlphaFold” (20), could now enable more reliable modeling of Wzy structures, facilitating studies on structure-function relationships. A good model for studying the relationship between Wzy sequences and its stereochemistry is the capsule of *Streptococcus pneumoniae* (pneumococcus). This gram-positive bacterium expresses at least 107 unique capsule types (serotypes) classed into 46 serogroups (21). Except for two, all pneumococcal capsules (*n* = 105) are synthesized via a Wzy-dependent pathway mediated by genes located in a single capsule synthesis (*cps*) locus (4). Wzy sequences for all capsule types have been determined (22) and are used for serotype identification (23). Furthermore, chemical structures of most Wzy-dependent capsule types (*n* = 96) have been determined: 80 serotypes exhibit β-configuration for the inter-RU glycosidic bond, while 16 display an α-configuration (21) (Fig. S1). Building on these findings, we harnessed the new computational power of AlphaFold 3 to predict the molecular structure of Wzy proteins across diverse pneumococcal serotype sequences. Outputs showed a striking difference between Wzy serotype homologs that fell into two structural-based categories, which paralleled either α-glycosidic or β-glycosidic linkages. In the absence of experimental structural data for a gram-positive Wzy protein, we present structural models of pneumococcal Wzy proteins and demonstrate their correlation with inter-RU glycosidic linkage stereochemistry.

## RESULTS

### Relationship among pneumococcal Wzy sequences

**Fig 1:**
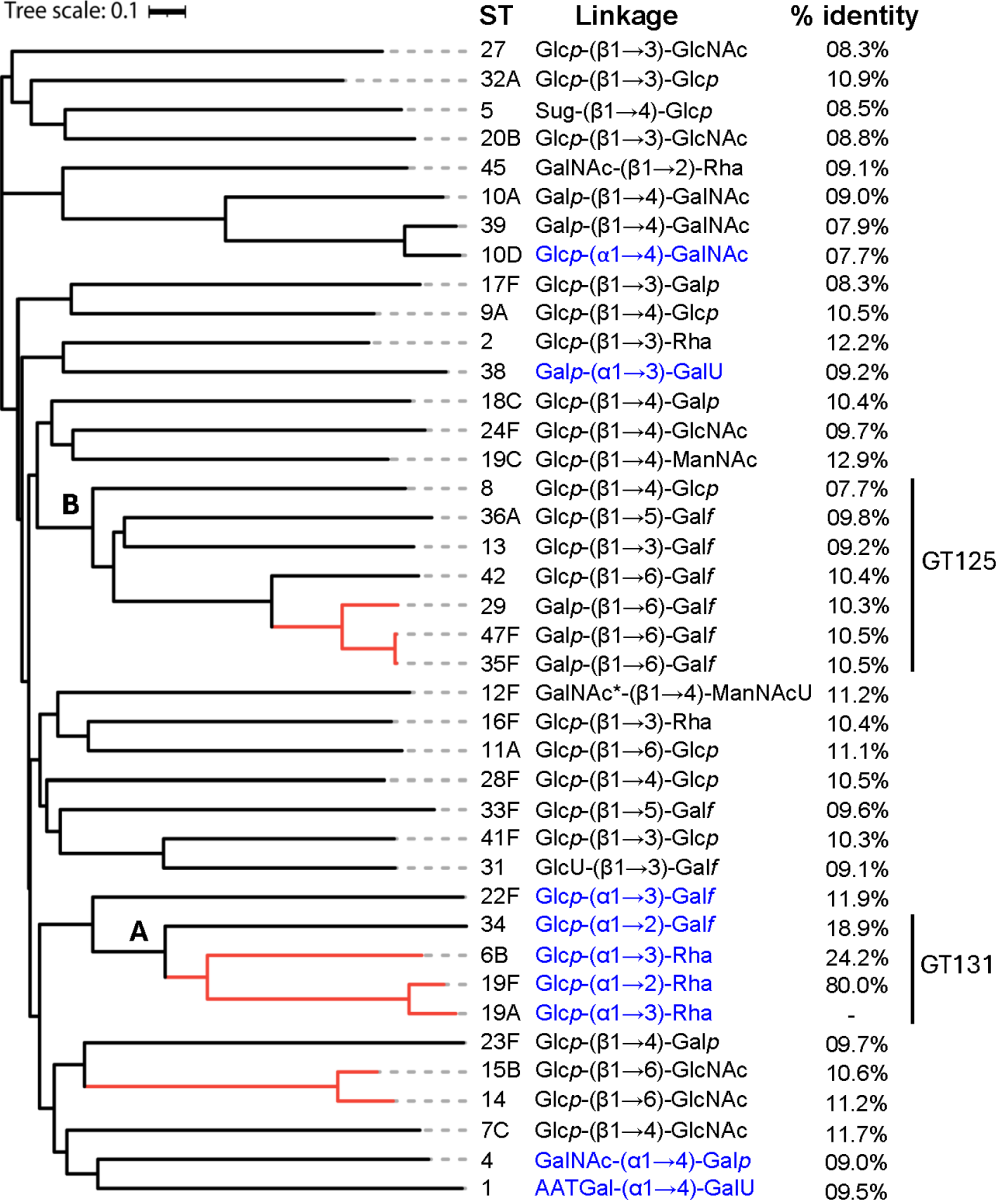
Phylogenetic analysis of pneumococcal Wzy. The phylogenetic tree was constructed using the amino acid sequences of 40 Wzy proteins, each representing a unique pneumococcal serogroup. As a notable exception, three serotypes from serogroup 19 were included in the analysis. Among these, serotypes 19A and 19F share an α-configuration but differ in their linkage positions, whereas 19C exhibits a β-configuration. Another exception was serogroup 10, wherein 10A catalyzes β- configuration and 10D catalyzes α-configuration. Clades containing highly similar Wzy proteins catalyzing identical glycosidic linkages are highlighted with red lines. Serotypes with α linkages are denoted in blue text, while those with β linkages remain unmarked. The % identity represents the amino acid sequence identity of all Wzy enzymes relative to 19A Wzy. Clusters A and B correspond to Wzy classified under the GT131 and GT125 CAZy families, respectively. The scale bar indicates evolutionary distance. ST, serotype. Asterisk (*) in serotype 12F linkage denotes a partial (25%) replacement of GalNAc with an atypical sugar residue called 2-acetamido-2,6-dideoxy-xylo-hexos-4-ulose (Sug). Glc*p*, glucopyranose; Gal*p*, galactopyranose; Gal*f*, galactofuranose; Rha, rhamnose; GlcNAc, N-acetylglucosamine; GalNAc, N-acetylgalactosamine; ManNAc, N-acetylmannosamine; ManNAcU, N-acetylmannosaminuronic acid; GlcU, glucuronic acid; GalU, galacturonic acid; AAT-Gal, 2-acetamido-4-amino-2,4,6-trideoxy-D-galactopyranoside.

To study the relationship among pneumococcal Wzys and their corresponding CPS products, we performed a phylogenetic analysis of 40 Wzy amino acid sequences (Fig. 1), each representing 1 of the 37 unique serogroups formed by the 96 structurally defined Wzy-dependent serotypes (Fig. S1). The selection was based on the observation that serotypes within a serogroup typically share the same inter-RU linkage configuration and cluster together phylogenetically (Fig. S2). The three exceptions were 19F, which we included for its prominent role in vaccine failures (24), as well as 10D and 19C, which exhibit distinct linkage configuration within their respective serogroups.

The phylogenetic analysis based on the genetic distance revealed no clear clustering patterns (Fig. 1), confirming the heterogeneity among Wzy. However, small clustering could be identified. For instance, Wzys from serotypes 19A and 6B, which catalyze identical α-Glc*p*-(1→3)-Rha linkages, clustered together, reflecting similarities in their donor/acceptor substrate recognition and linkage positions. Similarly, Wzys from serotypes 29, 35F, and 47F, responsible for β-Gal*p*-(1→6)-Gal*f* linkages, formed a distinct clade, as did Wzys from serotypes 14 and 15B, which catalyze β-Glc*p*-(1→6)-GlcNAc linkages.

The Carbohydrate-Active enZymes (CAZy) database (https://www.cazy.org/) classifies GTs into families based on sequence and functional similarity (25). However, the CAZy classification of Wzy proteins belonging to GT-C bacterial surface glycan polymerases has only recently been established (8). Our analysis identified multiple CAZy GT families corresponding to the distinct sequence clusters (Fig. 1). For instance, Cluster A, composed of Wzys from serotypes 6B, 19A, 19F, and 34, belongs to the GT-131 family, which is primarily associated with a retaining catalytic mechanism. Although Wzy from serotype 22F remains unclassified, it is also likely to be a part of the GT-131 family. Similarly, Cluster B consists of Wzys classified under the GT-125 family, which is mainly associated with an inverting catalytic mechanism. Notably, the CAZy classification for GT-C bacterial surface glycan polymerases like Wzy of pneumococci is not well established. Consequently, this association could not be determined for all Wzy clusters.

### Pneumococcal Wzy polymerases can be classified into two types based on their predicted 3D structures

**Fig 2:**
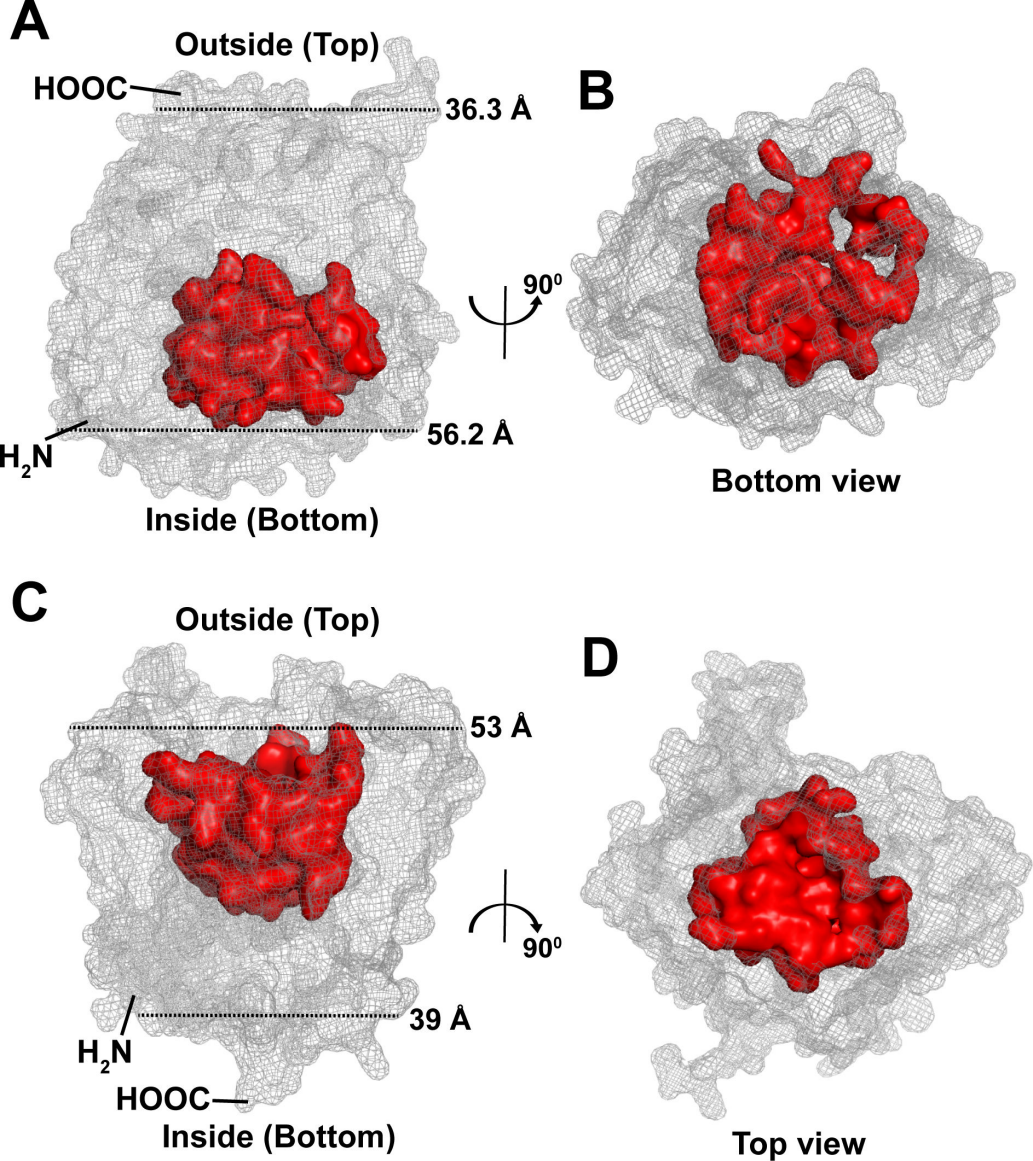
Predicted structural types of Wzy. (**A**) Type-A Wzy: represented by the serotype 19A Wzy, with the cavity (red) extending toward the cytoplasmic side (inside). C-terminus is located outside. The length of the Wzy at the top (36.3 Å) is measured between residues R428 and N444, while at the bottom (56.2 Å) between residues T2 and S124. (**B**) Bottom view of the serotype 19A Wzy structure obtained by a 90° rotation. (**C**) Type-B Wzy: represented by the serotype 14 Wzy, with the cavity (red) extending toward the extracellular side (outside). The C-terminus is positioned inside. The length of the Wzy at the top (53 Å) is measured between residues F71 and Q134, while at the bottom (39 Å) between L50 and R189. (**D**) Top view of the serotype 14 Wzy structure obtained by a 90° rotation. Both type-A and type-B Wzys have high confidence scores (average pLDDT ≥ 85; 95% CI: 85.86–86.01).

AlphaFold has recently emerged as a robust tool for predicting the three-dimensional (3D) structures of protein molecules, including membrane proteins (20). Using this approach, we obtained the predicted 3D structures of all 40 representative pneumococcal Wzy proteins with high confidence (average predicted local distance difference test [pLDDT] score ≥85; 95% confidence interval [CI]: 85.86–86.01). These Wzy models were characterized by an accessible “cavity” which may serve as the site for loading RUs during the polymerization process (26). Based on the location and orientation of the cavity and partially on C-terminus topology as predicted by the Orientation of Proteins in Membranes (OPM) computational tool (27, 28), Wzy predicted structures could be categorized into two distinct types: type-A and type-B (Fig. 2)

**Fig 3:**
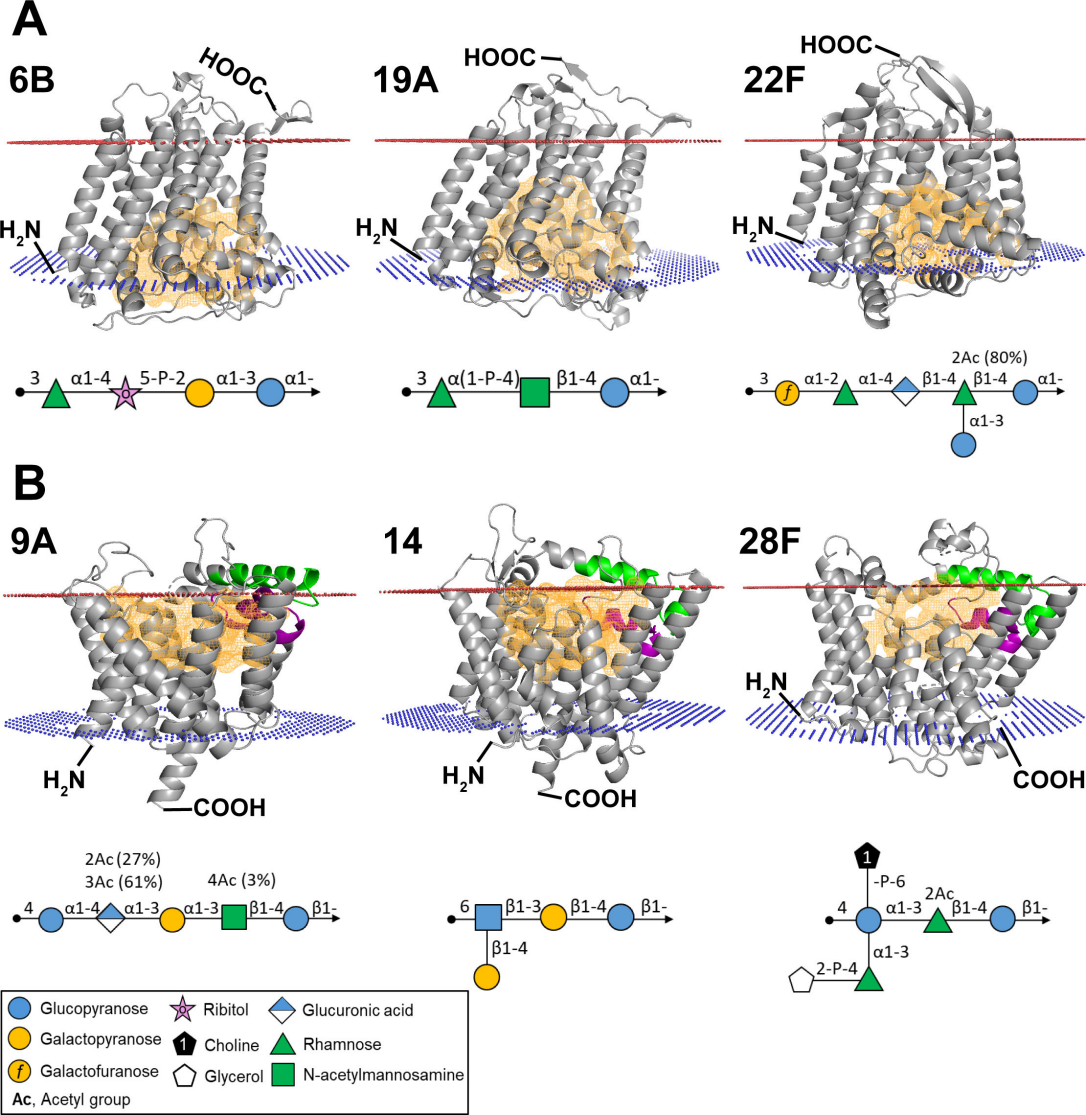
Correlation between pneumococcal Wzy structural types and inter-RU glycosidic bond configuration. (**A**) Type-A Wzy, representing serotypes 6B (GenBank accession: CR931639), 19A (GenBank accession: CR931675), and 22F (GenBank accession: CR931683). (**B**) Type-B Wzy, representing serotypes 9A (GenBank accession: CR931645), 14 (GenBank accession: CR931662), and 28F (GenBank accession: CR931693). Symbol nomenclature for glycan structures of CPS repeat units are shown below each corresponding Wzy structure. The orientation of Wzy within the membrane is depicted, showing the extracellular side (red mesh) and cytoplasmic side (blue mesh). In type-B Wzy structures, GN1 (green region) and GN2 (purple region) motifs are indicated. The cavity is shown (orange mesh) in both type-A and type-B Wzy.

Type-A Wzys featured a predicted cavity that extended toward the cytoplasm (Fig. 2 and 3), herein referred to as “bottom” open conformation. In contrast, type-B Wzys exhibited a predicted cavity oriented toward the extracellular interface (Fig. 2 and 3), referred to here as the “top” open conformation. Moreover, type-A Wzys have C-termini mostly located extracellularly, while the type-B Wzys have their C-termini typically positioned in the cytoplasm. Notably, all predicted Wzy structures shared a common feature of having their N-termini located in the cytoplasm, with none showing extracellular N-termini (Table 1).

**TABLE 1:** Pneumococcal Wzy structural types correlate with the RU linkage types

Wzy name^*d*^	GenBank accession	Wzy type	AlphaFold 3/OPM predicted features			Chemical linkage	Sequence traits	
			Open conformation	NTD	CTD		GN1^*a*^	GN2
5	CR931637	B	Top	Inside	Inside	β	Y	Y
7C	CR931642	B	Top	Inside	Inside	β	Y	Y
8	CR931644	B	Top	Inside	Inside	β	Y	Y
9A	CR931645	B	Top	Inside	Inside	β	Y	Y
11A	CR931653	B	Top	Inside	Inside	β	Y	Y
12F	CR931660	B	Top	Inside	Inside	β	Y	Y
13	CR931661	B	Top	Inside	Inside	β	Y	Y
14	CR931662	B	Top	Inside	Inside	β	Y	Y
15B	CR931664	B	Top	Inside	Inside	β	Y	Y
16F	CR931668	B	Top	Inside	Inside	β	Y	Y
17F	CR931670	B	Top	Inside	Inside	β	Y	Y
18C	CR931673	B	Top	Inside	Inside	β	Y	Y
19C	CR931677	B	Top	Inside	Inside	β	Y	Y
23F	CR931685	B	Top	Inside	Inside	β	Y	Y
24F	CR931688	B	Top	Inside	Inside	β	Y	Y
27	CR931691	B	Top	Inside	Inside	β	N	Y^*c*^
28F	CR931693	B	Top	Inside	Inside	β	Y	Y
29	CR931694	B	Top	Inside	Inside	β	Y	N
31	CR931695	B	Top	Inside	Inside	β	Y	Y
32A	CR931696	B	Top	Inside	Inside	β	Y	Y
33F	CR931702	B	Top	Inside	Inside	β	Y	N
35F	CR931707	B	Top	Inside	Inside	β	Y	Y^*b*^
36A	CR931708	B	Top	Inside	Inside	β	Y	Y^*c*^
41F	CR931714	B	Top	Inside	Inside	β	Y	Y
42	CR931715	B	Top	Inside	Inside	β	Y	N
47F	CR931721	B	Top	Inside	Inside	β	Y	Y^*b*^
2	CR931633	B	Top	Inside	Outside	β	Y	Y
10A	CR931649	B	Top	Inside	Outside	β	Y	Y
39	CR931711	B	Top	Inside	Outside	β	N	Y
1	CR931632	B	Top	Inside	Inside	α	N	N
4	CR931635	B	Top	Inside	Inside	α	N	N
38	CR931710	B	Top	Inside	Inside	α	N	N
6B	CR931639	A^*e*^	Bottom	Inside	Outside	α	N	N
10D	ERR051587	A^*e*^	Bottom	Inside	Outside	α	N	N
19A	CR931675	A^*e*^	Bottom	Inside	Outside	α	N	N
19F	CR931678	A^*e*^	Bottom	Inside	Outside	α	N	N
22F	CR931682	A^*e*^	Bottom	Inside	Outside	α	N	N
34	CR931703	A^*e*^	Bottom	Inside	Outside	α	N	N
20B	JQ653093	A^*e*^	Bottom	Inside	Inside	β	N	N
45	CR931718	A^*e*^	Bottom	Inside	Inside	β	N	N

^*a*^ "Y” denotes the presence of the motif, while “N” denotes its absence.

^*b*^ GN2 motif in Wzy contains a GX_4_G region instead of GX_2_G.

^*c*^ Wzy possesses a weak GN2 motif.

^*d*^ Pneumococcal serotype from which Wzy amino acid sequence was extracted. NTD, N-terminal domain; CTD, C-terminal domain.

^*e*^ It is possible that type-A Wzys may exhibit reverse topological predictions, as OPM-based predictions for type-A Wzys may not fully reflect the native orientation. For instance, 6B Wzy may have “Top” open conformation, “outside” NTD, and “inside” CTD.

Although OPM is a widely used computational tool for predicting the orientation of proteins in membranes (27, 28), our in-silico docking analyses of Wzy with biosynthetic partners such as CpsC/CpsD suggest that the orientation predicted by OPM for type-A Wzys may not be entirely accurate (data not shown). CpsC and CpsD are components of the bacterial tyrosine kinase system that regulates CPS synthesis post-transcriptionally. In pneumococcus, CpsC is a transmembrane protein that enables autophosphorylation of CpsD, a cytoplasmic tyrosine kinase (4, 29). Both proteins belong to the PCP2b family of polysaccharide co-polymerases (PCPs) and are implicated in the regulation of capsule polymer length, presumably by interacting with the Wzy polymerase (29). While this does not affect the overall classification of Wzys into type-A and type-B, it implies that type-A Wzys may instead possess an extracellular N-terminus and an intracellular C-terminus, with the predicted cavity exhibiting “top” open conformation.

### Wzy predicted 3D structural types correlate with the glycosidic bond configuration in pneumococci

The structural classification of pneumococcal Wzy polymerases into type-A and type-B shows a strong correlation with the inter-RU glycosidic bond configuration (i.e., α or β) of capsular polysaccharides. Type-A Wzys are predominantly associated with α-glycosidic bonds (*P* > 0.05), whereas type-B Wzys primarily catalyze β-glycosidic bonds (*P* < 0.001), as illustrated in Fig. 3 and Table 1. However, exceptions to this trend were observed, as a small subset of the Wzys deviated from the expected structural-glycosidic bond configuration relationship (Table 1). For example, despite being classified as type-B, Wzys from serotypes 1, 4, and 38 catalyze α-glycosidic bond configurations. Similarly, structural anomalies were also identified in type-A Wzys, such as those from serotypes 20 and 45, which catalyze β-glycosidic bond configuration.

### Wzy 3D structural types can be distinguished based on the conserved sequence motifs

**Fig 4:**
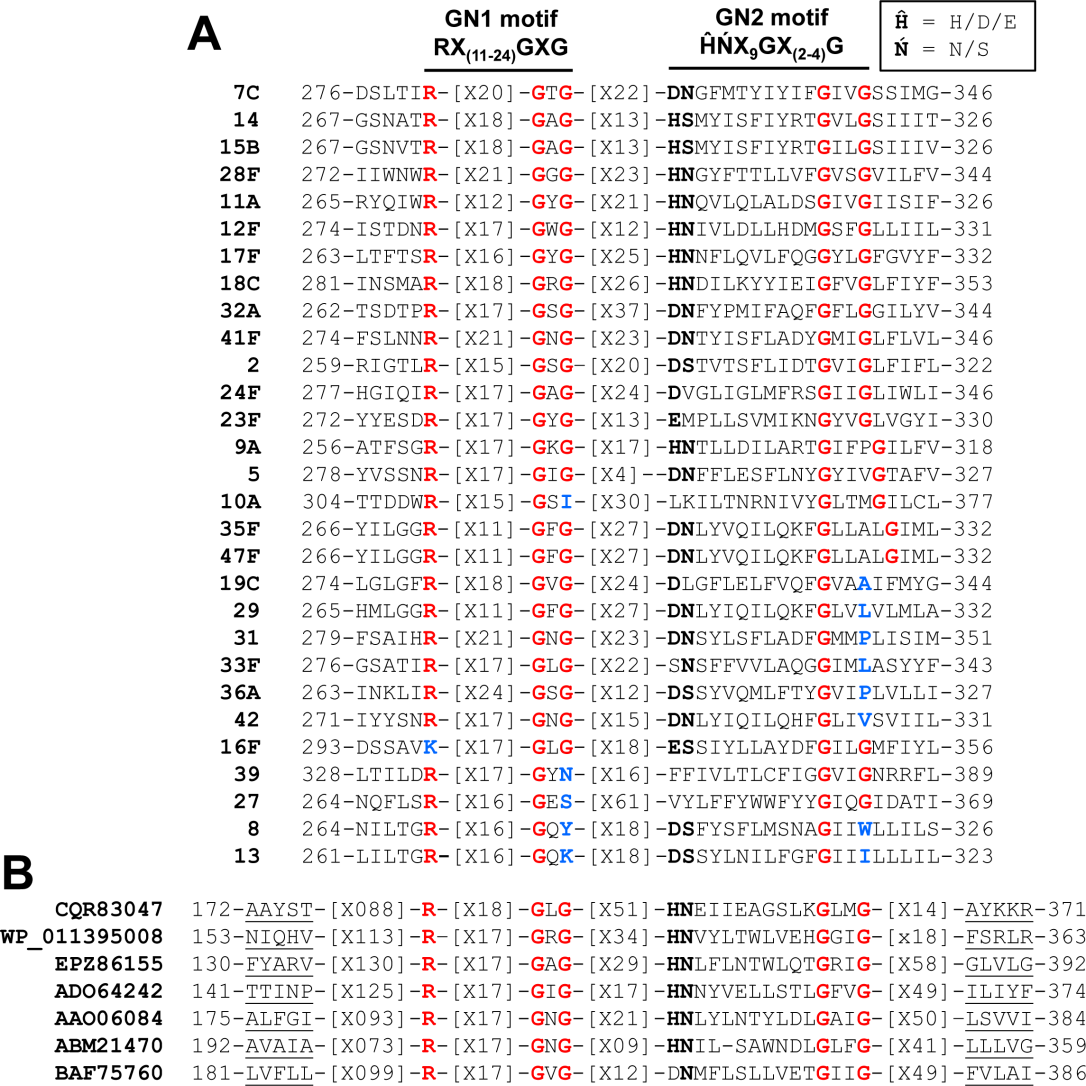
Identification of location-specific conserved sequence motifs. (**A**) Alignment of non-contiguous type-B Wzy sequences from multiple pneumococcal serotypes. GN1 and GN2 motifs are denoted as bold letters on top of the alignment. Three-digit numbers on the left and right sides of the alignment indicate the regions containing GN1 and GN2 motifs. Residue conservation is represented as follows: red for highly conserved residues, blue for divergent residues. The letter “X” denotes any residue, with the associated number specifying the count of such residues. A key explaining the special characters in the GN2 motif is provided alongside the motif at the top. The Wzy names are listed in the first column on the left side of the alignment, and their corresponding GenBank accession numbers are listed in Table 1. (**B**). Alignment of the annotated Wzy_C domain sequences, associated with the PF04932 and COG3307, from O-antigen ligases/polymerases across multiple gram-negative species. The alignment shows the presence of GN1 and GN2 motifs within the Wzy_C domain, with its starting and ending residues underlined. GenBank accession numbers for each protein sequence are listed in the first column on the left. The analyzed sequences correspond to the following species: *Escherichia coli* (CQR83047), *Hahella chejuensis* (WP_011395008), *Burkholderia cenocepacia* (EPZ86155), *Vibrio vulnificus* (ADO64242), *Salmonella phage epsilon15* (AAO06084), *Pseudomonas aeruginosa* (ABM21470), and *Klebsiella pneumoniae* (BAF75760).

To simplify the classification of Wzy proteins, we sought to identify type-specific conserved amino acid sequence motifs that could serve as molecular signatures, complementing structural distinctions predicted by AlphaFold and glycosidic bond configurations. Attempts to identify a conserved motif unique to type-A Wzys failed. However, two amino acid motifs, herein referred to as “GN1 motif” (RX_(11-24)_GXG) and “GN2 motif” (ĤŃX_9_GX_(2-4)_G), were found to be useful markers for distinguishing type-A from type-B (Fig. 4A). Type-A Wzys lacked both motifs (*P* < 0.01), whereas most type-B Wzys possessed at least one (*P* < 0.001) (Table 1).

Interestingly, three type-B Wzys (serotypes 1, 4, and 38) lacked both motifs, and all were associated with an α-glycosidic bond. Conversely, two type-A Wzys (serotypes 20 and 45) associated with β-glycosidic bond also lacked these motifs (Table 1). Although the correlation is not absolute, these motifs provide a promising means of differentiating the two Wzy types based on sequence analysis, with a stronger association observed between the motifs and glycosidic bond configuration.

### GN1 and GN2 motifs are critical for Wzy polymerase activity

To determine the functional importance of the GN1 and GN2 motifs, we performed targeted genetic manipulations in the *wzy*_23F_ of MBO494, a pneumococcal strain that co-expresses 23A (under native *cps* locus) and 23F (*wzy*_23F_ under Tet-on inducible promoter) capsules (Table 2). Serotypes 23A and 23F are structurally identical except in the Wzy-mediated linkage. This novel expression system enables selective manipulation of *wzy*_23F_ without interrupting capsule biosynthesis by preventing lethal depletion of Und-P resulting from the loss of *wzy*.

**TABLE 2:** Genetic manipulation of GN1 and GN2 motifs

Mutant	Genetic manipulation in Ptet-*wzy*_23F_, genotype*^a^*	Capsule expression by flow cytometry	
		23A (FS23c)	23F (Hyp23FG3)
MNG116	WT (TIGR4, 23A *cps* locus)	Yes	No
MBO494	MNG116, Ptet-*wzy*_23F_	Yes	Yes
MBO495	ΔR277 to G297	Yes	No
MBO498	R277A	Yes	Yes
MBO499	G295A	Yes	Yes
MBO500	G297A	Yes	Yes
MBO501	R277A + G295A	Yes	Yes
MBO502	R277A + G297A	Yes	Yes
MBO503	G295A + G297A	Yes	Yes
MBO504	R277A + G295A + G297A	Yes	Yes
MBO496	ΔE311 to G325	Yes	No
MBO505	G322A	Yes	No
MBO506	G325A	Yes	Yes
MBO507	G322A + G325A	Yes	No

^*a*^ Genetic manipulations were performed on the *wzy*_23F,_ which is expressed under Tet-On inducible promoter (Ptet-*wzy*_23F_, tetracycline-dependent) in the MBO494 strain. Symbol (Δ) indicates deletion. WT, wild type. Hyp23FG3 is a serotype 23F-specific monoclonal antibody, and FS23c is a serotype 23A-specific polyclonal rabbit antiserum. Strains MBO495 and MBO498-MBO504 are GN1 motif-targeted mutants, while MBO496 and MBO505-507 are GN2 motif-targeted mutants. MNG116 is a TIGR4_Δ_*_cps_* pneumococcal strain carrying 23A *cps* locus from strain SSISP23A. MBO494 is a pneumococcal strain that co-expresses serotype 23A (under native *cps* locus) and serotype 23F (*wzy*_23F_ under Tet-on inducible promoter).

Deletion of either GN1 or GN2 motifs in Wzy_23F_ resulted in complete loss of 23F capsule expression, indicating a critical role of these motifs in polymerase function (Table 2). To further dissect the contribution of individual residues within these motifs, we performed alanine-scanning mutagenesis by systematically substituting each residue with alanine. Interestingly, none of the single/multiple alanine substitutions in GN1 abolished capsule expression, suggesting some degree of residue-level tolerance within the motif. In contrast, a single amino acid substitution in GN2 (G322A) was sufficient to abolish Wzy function, mirroring the complete deletion phenotype (Table 2). These findings collectively demonstrate that both GN1 and GN2 motifs are essential for Wzy activity. However, further studies are required to confirm that the observed phenotype is due to impaired catalytic activity rather than protein misfolding or instability.

## DISCUSSION

To our knowledge, this is the first study to establish a correlation between predicted 3D molecular architecture of pneumococcal Wzys and the glycosidic linkages they catalyze, thereby providing new insights into its structure-function relationship. By combining AlphaFold 3-predicted structural models, OPM-based membrane orientation, and sequence analysis, we identified two distinct structural types of Wzy—type-A and type-B—with different topologies that may influence substrate accessibility and orientation during glycan polymerization. Interestingly, both types showed a strong correlation with their respective glycosidic bond configurations (i.e., α or β; retaining or inverting the anomeric configuration). The predicted Wzy structural differences likely facilitate distinct interactions with diverse donor/acceptor substrates, potentially involving different chemical reaction mechanisms that influence the final glycosidic bond configuration (7, 8).

In pneumococci, AlphaFold-predicted Wzy structures (type-A or type-B) generally correlated with inter-RU glycosidic bond configurations (α or β), though a few exceptions suggest potential complexities. Discrepancies may arise from inaccuracies in glycosidic bond annotations, as some structures were determined decades ago using low-resolution analytical techniques (30, 31). Interestingly, we identified a discrepancy between AlphaFold-predicted models and the inter-RU glycosidic bond configurations for serotypes 36A and 36B. While the AlphaFold model classified them as type-B, initial biochemical analysis determined α-glycosidic bond configuration. Re-examination of their biochemical structures using NMR data led us to revise their Wzy-mediated linkage configurations from α to β (32). Another possibility could be the presence of multiple Wzy homologs, as observed in serotype 33G, where a Wzy homolog (Orf2) acquired from other streptococci likely functions as the polymerase (33). Similarly, bacteriophages may also influence Wzy-mediated glycosidic linkages. In *Pseudomonas aeruginosa* serotypes O2 and O16, a phage-mediated transfer of a phage-*wzy* located ectopically could override the native Wzy, thereby altering linkage types (34, 35).

Pneumococcal Wzy molecules analyzed in this study range from 400 to 477 amino acids. Their high sequence variability has hindered efforts to annotate Wzy or classify them as type-A or type-B. To address this, we defined two conserved motifs—GN1 and GN2—present in most type-B Wzys, which show a stronger association with β-configuration. Notably, GN1 and GN2 motifs are location-specific, appearing toward the C-terminal region (~250 amino acids onward). These motifs belong to the PF04932 and COG3307 domains, which are prominently associated with the conserved O-antigen ligase region, known as Wzy_C domain (36–38) (Fig. 4B). While the identification of GN1 and GN2 motifs enables rapid sequence mining to distinguish type-A and type-B Wzys, the sequence variation precluded us from precisely defining the Wzy_C homolog in pneumococcal Wzy. However, the presence of these motifs could serve as an indicator of a Wzy_C domain in the given Wzy protein sequence.

The Wzy_C domain is present within the distal region of O-antigen ligases like WaaL in gram-negative bacteria (37, 38) and is associated with inverting glycosyltransferase activity, catalyzing the linkage of O-antigen to lipid A-core (39, 40). Interestingly, the Wzy_C domain has been found conserved in 82% of Wzy proteins associated with β-linked O-antigen structures but is absent in those linked to α-configurations (41). While the presence of the Wzy_C domain suggests its potential role in β-polymerase activity, its exact contribution in Wzy may differ from WaaL, as both proteins differ significantly in structure and substrate specificity (3, 42, 43), making direct functional parallels unclear.

Although our analysis focused on pneumococcal capsules, our findings may potentially extend to other bacterial species, including gram-negative bacteria that produce Wzy-dependent glycopolymers. However, the extent to which the relationship between Wzy structure and glycosidic bond configuration in other species aligns with that of pneumococci remains unclear. The differences may arise from variations in glycan synthesis mechanisms and the involvement of distinct biosynthetic partners, such as PCPs that interact with Wzy. For example, pneumococci utilize Wzd (aka. CpsC) as the PCP for capsule synthesis (29), whereas gram-negative bacteria such as *Klebsiella* employ Wzc for capsule synthesis (44). Similarly, in O-antigen synthesis, *Shigella* and *Salmonella* rely on Wzz as the PCP (45–47).

While this study provides valuable insights, it has some limitations. To date, no high-resolution structural data—such as X-ray crystallography or cryo-EM—are available for full-length Wzy homologs from any bacterial species, leaving their tertiary structures largely undefined and posing a challenge to fully elucidating their structure-function relationship. Although AlphaFold 3 predictions in conjunction with OPM offer a useful starting point for exploring Wzy conformations and potential mechanisms, experimental validation is warranted. For instance, OPM-based orientation predictions for pneumococcal Wzys, particularly type-A, may not be fully accurate. Thus, experimental studies are needed to confirm membrane orientation and topology of both Wzy types.

Additionally, structural determination of Wzy in complex with native polyisoprenyl pyrophosphate-linked substrates would reveal substrate binding sites and enable detailed polymerization kinetics. Capturing Wzy in different conformational states, via cryo-EM or molecular dynamics, could clarify the mechanisms of α-glycosidic and β-glycosidic bond formation. Furthermore, high-resolution structural characterization of Wzy-PCP complexes, coupled with biochemical and molecular studies, will be crucial to uncovering the molecular basis of their interactions. Lastly, further studies examining additional Wzy proteins from other bacterial species are needed to determine the broader applicability of this structural-glycosidic bond relationship in bacterial surface glycopolymers with experimental evidence.

## MATERIALS AND METHODS

### Extraction and multiple alignment of Wzy amino acid sequences

Wzy amino acid sequences were retrieved from the reference *cps* loci available in NCBI GenBank (https://www.ncbi.nlm.nih.gov/) and evaluated for correct translational frames. Amino acid sequences of Wzy proteins were extracted using Geneious Prime v2019.2 (Biomatters). Multiple sequence alignments were conducted using the MUSCLE alignment tool integrated into Geneious Prime, enabling the comparison of sequences and identification of conserved regions.

### Phylogenetic analysis

To investigate the sequence diversity among pneumococcal Wzy proteins, we used the amino acid sequences from 40 pneumococcal Wzys representing 37 individual serogroups. Phylogenetic analyses were conducted based on genetic distance using Geneious Prime v2019.2 (Biomatters). Full-length amino acid sequences were aligned by MAFFT (multiple alignment using fast Fourier transform), and the tree was constructed based on the neighbor-joining method using the Tamura-Nei genetic distance model with 1,000 bootstrap replicates. Display and manipulation of the phylogenetic tree (Newick format) were performed using the online tool Interactive Tree of Life v7.0 (https://itol.embl.de).

### Generation and visualization of AlphaFold 3D models

The amino acid sequences of Wzy proteins were extracted from the relevant databases and directly deposited into the AlphaFold 3 server (20). The server’s predictive algorithm was used to generate 3D structural models. The generated models were exported and subsequently visualized using the Biovia Discovery Studio v24.1.0 or PyMOL v3.0.4. To identify the orientation of the Wzy proteins within the lipid bilayer, the predicted models (PDB files) were analyzed using the OPM web-based PPM server (https://opm.phar.umich.edu/ppm_server) (27). Annotations of key functional residues and regions/motifs were made in Discovery Studio. To assess the reliability of AlphaFold-predicted Wzy structures, we utilized the pLDDT score, which evaluates the confidence of individual residue-level predictions on a scale of 0–100 (48). The average pLDDT for each Wzy type was calculated by averaging per-residue confidence scores across the full-length sequence. The confidence categories were defined as follows: very high confidence: 90–100; high confidence: 70–89; low confidence: 50–69; very low confidence: <50.

### Genetic manipulation

Primers used in this study are listed in Table S1. The recombinant strain MBO494, which co-expresses serotype 23A and 23F capsules, was generated by transforming a ~4.7 kb Ptet-*wzy*_23F_ DNA fragment into the *bgaA–spr0566-68* insertion site of MNG116 (pneumococcal TIGR4_Δ_*_cps_* strain carrying 23A *cps* locus, GenBank accession no: CR931683) (Table 2). The Ptet-*wzy*_23F_ construct was generated by fusing the Tet-On inducible promoter from plasmid pONC6.8 (GenBank accession no: MZ054181) (49, 50) with the 1,194 bp *wzy*_23F_ gene (PCR-amplified from EMC23F genomic DNA, a serotype 23F strain; GenBank accession no: JAXIJT000000000) using overlap extension PCR (49). Subsequently, site-directed mutagenesis targeting single or multiple residues within the GN1 and GN2 motifs of *wzy*_23F_ was then performed using MBO494 as the template and MNG116 as the recipient. Mutant strains were selected on THY agar containing 15 µg/mL trimethoprim. Expression of *wzy*_23F_ was induced by culturing the bacteria in THY broth with 100 ng/mL anhydrous tetracycline (Abcam, Cambridge, UK) at 37°C for 45 minutes.

### Flow cytometry analysis

The phenotypic capsule expression on the bacterial surface was determined by flow cytometry as previously described (51–53). Briefly, frozen bacterial stocks were thawed, washed, and incubated in flow cytometry serotyping assay (FCSA) buffer (phosphate-buffered saline, 3% fetal bovine serum, 0.1% NaN_3_) containing 1:160 dilution of our in-house Hyp23FG3 (serotype 23F specific) monoclonal antibody and 1:9,000 factor serum 23c (FS23c), a polyclonal rabbit antiserum (Statens Serum Institut, Copenhagen, Denmark), for 30 minutes at 4°C. After washing, bound immunoglobulin (Ig) was stained with 1:1,000 dilution of phycoerythrin-labeled anti-mouse/anti-rabbit Ig antibody (Southern Biotech, Birmingham, AL, USA) in FCSA buffer, and detected by flow cytometry using BD Accuri C6 Plus (BD Biosciences, Franklin Lakes, USA) and FCS Express software (Pasadena, USA).

### Statistical analysis

A two-tailed binomial test was conducted to evaluate the statistical significance of the correlation between AlphaFold-predicted structural types and glycosidic bond configuration or the conservation of sequence motifs. The test calculated the probability of obtaining the observed outcomes under the null hypothesis (probability = 0.5), summing the probabilities in both tails of the binomial distribution. Statistical significance was set at *P* < 0.05, and calculations were performed using SciPy (Python). The 95% CI for the average pLDDT score was calculated using the *t*-distribution, based on the mean, sample size, and sample standard deviation of pLDDT values across all (*n* = 40) predicted Wzy structures.

## Data Availability

All data generated or analyzed during this study are included in the manuscript. All nucleotide or amino acid sequences analyzed have been provided with their respective GenBank accession numbers. Additional supporting data or materials not included in the article are available from the corresponding author upon request.

## SUPPLEMENTAL MATERIAL

Supplemental material spectrum.00328-25-s0001.pdf Figures S1 and S2; Table S1.

ASM does not own the copyrights to Supplemental Material that may be linked to, or accessed through, an article. The authors have granted ASM a non-exclusive, world-wide license to publish the Supplemental Material files. Please contact the corresponding author directly for reuse.
