# Supplementary material for: Wzy 3D structural models correlate with inter-repeat unit glycosidic bond configuration in pneumococcal capsule polysaccharides
Source: Microbiol Spectr. 2025 Sep 3;13(10):e00328-25. doi: 10.1128/spectrum.00328-25 (PMC12502699; doi:10.1128/spectrum.00328-25)
Supplement: Supplemental material — Figures S1 and S2; Table S1. [file spectrum.00328-25-s0001.pdf]

Supplementary material for:

Wzy 3D structural models correlate with inter-repeat unit glycosidic bond configuration in pneumococcal capsule polysaccharides

Feroze A. Ganaie[1,#], Melissa B. Oliver[1], Jamil S. Saad[2], David G. Glanville[1], Andrew T. Ulijasz[1], Moon H. Nahm[1]

[1]Division of Pulmonary, Allergy and Critical Care, Department of Medicine, The University of Alabama at Birmingham, Birmingham, AL, USA

[2]Department of Microbiology, The University of Alabama at Birmingham, Birmingham, AL, USA

Running title: Pneumococcal Wzy can be classified into two types

[#] Correspondence to Feroze A. Ganaie, fganaie@uabmc.edu

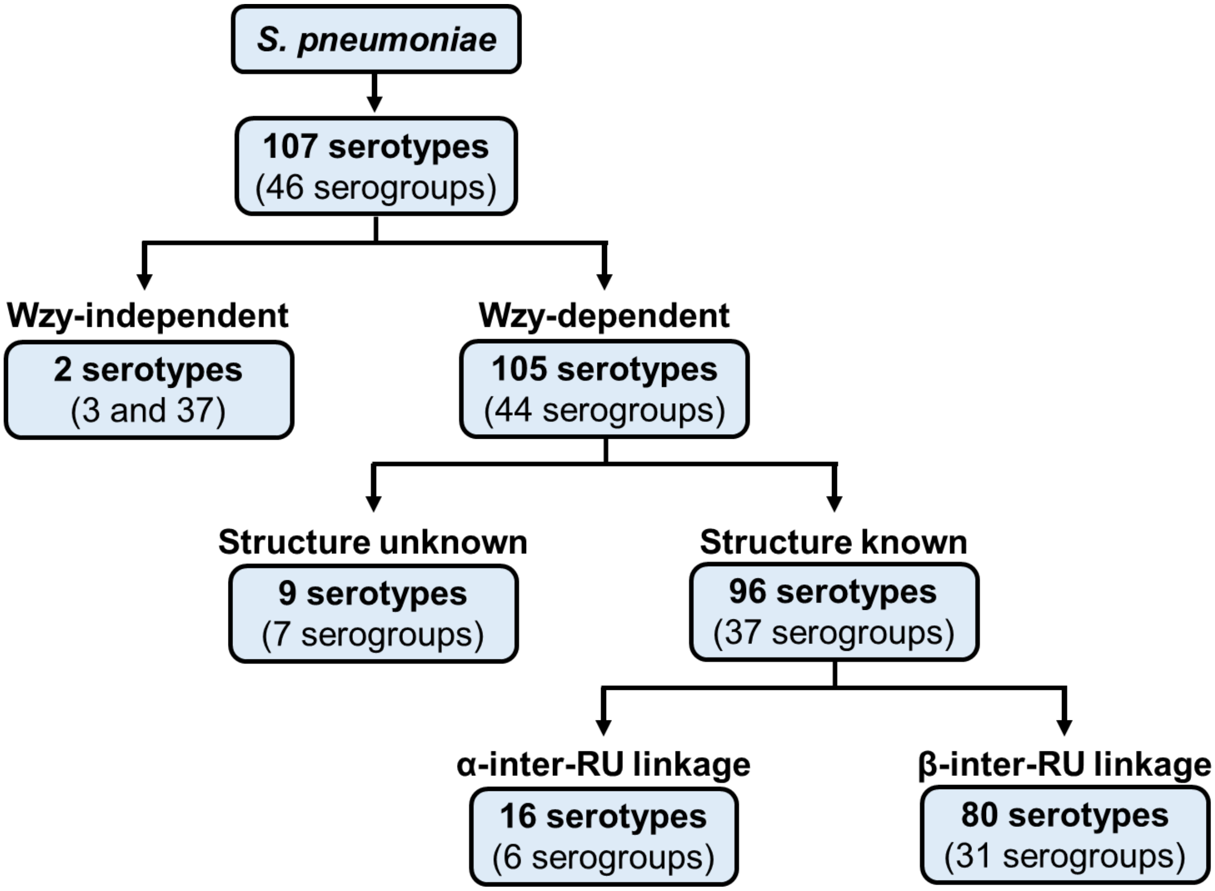

Fig. S1. Classification of pneumococcal serotypes/serogroups. The flow diagram shows how pneumococcal serotypes/serogroups are classified based on the Wzy-dependent pathway, biochemical structure, and Wzy-mediated inter-repeat unit glycosidic linkages.

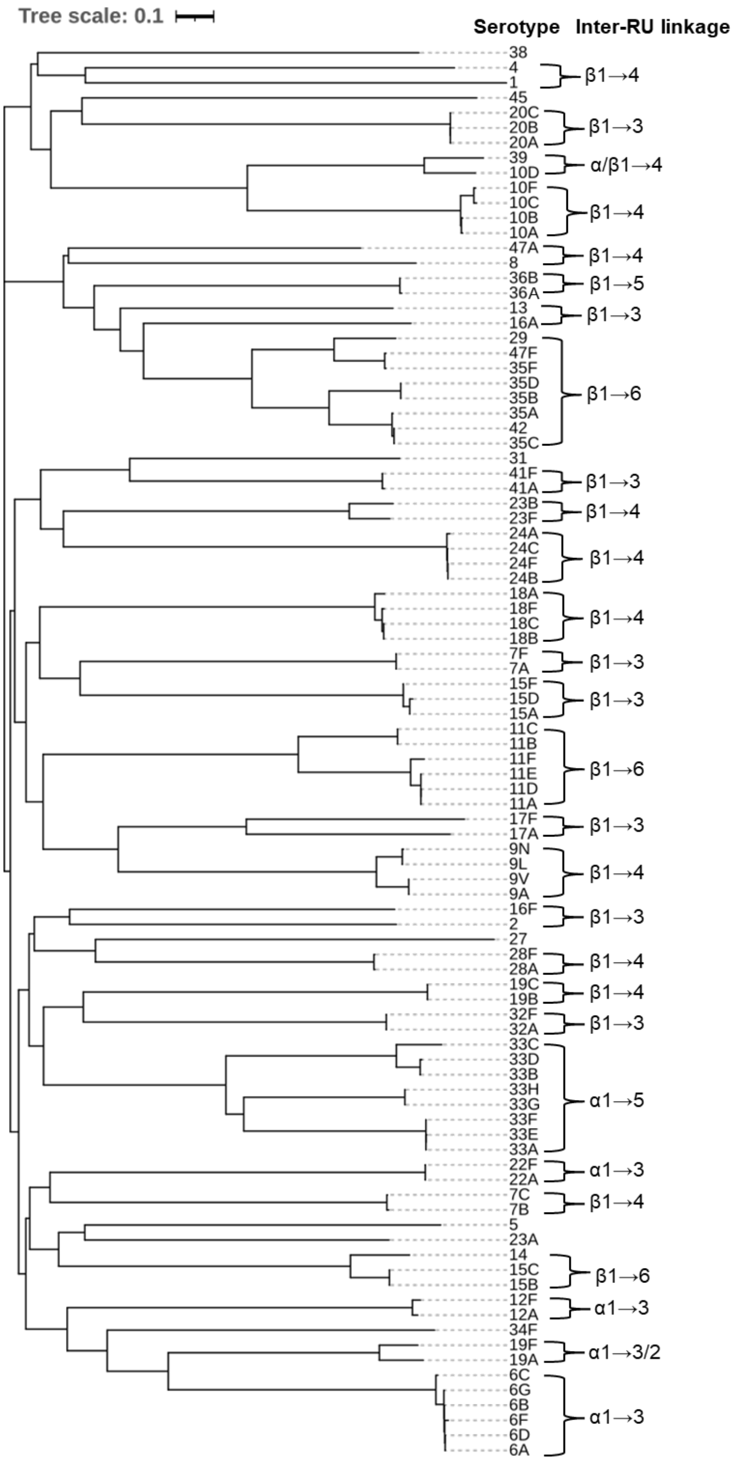

Fig. S2. Phylogenetic analysis of 94 pneumococcal Wzy proteins.

The tree was generated using amino acid sequences from Wzys of 94 serotypes with known capsule polysaccharide chemical structures. Serotypes 7D and 6H were excluded due to unavailability of Wzy sequences. Inter-repeat unit glycosidic linkages are indicated only for Wzys that form phylogenetic clusters.

Table S1. Primers used in this study

Strain	Target mutation	Fragment	Primer	Oligonucleotide sequence	Size (bp)
MBO494	Creating construct Ptet-*wzy*$_{23F}$	494_F1	3838	TCTTCTGGCGTGCAGGATTCTTATTAACC	1914
			31483	CAAATTATTAATTTTTATAGTCACTTCAAAATCCTCCTTACTAGAGCAAGGATCTG	
		494_F2	51719	CTAGTAAGGAGGATTTTGAAGTGACTATAAAAATTAATAATTTGTTTTTTGT	1232
			31469	CTAGTCCAAGCTCACAAGTTACTTTCTGTTATTTACTAATTTTATAT	
		494_F3	51703	GTAAATAACAGAAAGTAACTTGTGAGCTTGGACTAGAAAAAAACTTCAC	1702
			5980	GCTGTCAAAGCCAAAGTCATGAGAT	
MBO495	ΔGN1 (R277 to G297)	495_F1	3838	TCTTCTGGCGTGCAGGATTCTTATTAACC	2736
			31470	CACACTTCTAATATTGTGATCTGATTCATAATATTTAAAAAAATTCAC	
		495_F2	51704	TAAATATTATGAATCAGATCACAATATTAGAAGTGTGTTAGGTTGGGATG	2006
			5980	GCTGTCAAAGCCAAAGTCATGAGAT	
MBO496	ΔGN2 (E311 to G325)	496_F1	3838	TCTTCTGGCGTGCAGGATTCTTATTAACC	2840
			31471	CTATAATGTAACCAACTAATACCGTTCCATCCCAACCTAACACACTTCT	
		496_F2	51705	GGTTGGGATGGAACGGTATTAGTTGGTTACATTATAGTGCTGTTTAAG	1921
			5980	GCTGTCAAAGCCAAAGTCATGAGAT	
MBO497	ΔGN1 + ΔGN2	497_F1	3838	TCTTCTGGCGTGCAGGATTCTTATTAACC	2746
			31472	CCCAACCTAACACACTTCTAATATTGTGATCTGATTCATAATATTTAAAAAAATTCAC	
		497_F2	51706	GAAGTGTGTTAGGTTGGGATGGAACGGTATTAGTTGGTTACATTATAGTGCTGTTTAAG	1932
			5980	GCTGTCAAAGCCAAAGTCATGAGAT	
MBO498	R277A	498_F1	3838	TCTTCTGGCGTGCAGGATTCTTATTAACC	1739
			31473	CCCAAAAAACAAATGAAATGCATCTGATTCATAATATTTAAAAAAATTCACAAC	
		498_F2	51707	TAAATATTATGAATCAGATGCATTTCATTTGTTTTTTGGGGATGCTGAATTAGC	2069
			5980	GCTGTCAAAGCCAAAGTCATGAGAT	
MBO499	G295A	499_F1	3838	TCTTCTGGCGTGCAGGATTCTTATTAACC	1792
			31474	CTAATATTGTGTCCATATGCCTTCGTCGTATTTCCAAAGGCTAATTCAGC	

		499_F2	51708	CTTTGGAAATACGACGAAGGCATATGGACACAATATTAGAAGTGTGTTAGGTTG	2015
			5980	GCTGTCAAAGCCAAAGTCATGAGAT	
MBO500	G297A	500_F1	3838	TCTTCTGGCGTGCAGGATTCTTATTAACC	2799
			31475	CACACTTCTAATATTGTGTGCATAACCCTTCGTCGTATTTCCAAAGGCTAATTC	
		500_F2	51709	ATACGACGAAGGGTTATGCACACAATATTAGAAGTGTGTTAGGTTGGGATG	2007
			5980	GCTGTCAAAGCCAAAGTCATGAGAT	
MBO501	R277A + G295A	501_F1	3838	TCTTCTGGCGTGCAGGATTCTTATTAACC	2753
			31480	GCTAATTCAGCATCCCCAAAAAACAAATGAAATGCATCTGATTCATAATATTTAAAAAAATTC	
		501_F2	51714	TGGGGATGCTGAATTAGCCTTTGGAAATACGACGAAGGCATATGGACACAATATTAGAAGTGTGTTAG	2033
			5980	GCTGTCAAAGCCAAAGTCATGAGAT	
MBO502	R277A + G297A	502_F1	3838	TCTTCTGGCGTGCAGGATTCTTATTAACC	2753
			31480	GCTAATTCAGCATCCCCAAAAAACAAATGAAATGCATCTGATTCATAATATTTAAAAAAATTC	
		502_F2	51715	TGGGGATGCTGAATTAGCCTTTGGAAATACGACGAAGGGTTATGCACACAATATTAGAAGTGTGTTAGGTTGGG	2033
			5980	GCTGTCAAAGCCAAAGTCATGAGAT	
MBO503	G295A + G297A	503_F1	3838	TCTTCTGGCGTGCAGGATTCTTATTAACC	2799
			31476	CACACTTCTAATATTGTGTGCATATGCCTTCGTCGTATTTCCAAAGGCTAATTCAGCATC	
		503_F2	51710	CTTTGGAAATACGACGAAGGCATATGCACACAATATTAGAAGTGTGTTAGGTTGGGATGG	2015
			5980	GCTGTCAAAGCCAAAGTCATGAGAT	
MBO504	R277A + G295A + G297A	504_F1	3838	TCTTCTGGCGTGCAGGATTCTTATTAACC	2753
			31480	GCTAATTCAGCATCCCCAAAAAACAAATGAAATGCATCTGATTCATAATATTTAAAAAAATTC	
		504_F2	51716	TGGGGATGCTGAATTAGCCTTTGGAAATACGACGAAGGCATATGCACACAATATTAGAAGTGTGTTAGGTTGGG	2033
			5980	GCTGTCAAAGCCAAAGTCATGAGAT	
MBO505	G322A	505_F1	3838	TCTTCTGGCGTGCAGGATTCTTATTAACC	2874

			31477	ACCAACTAACCCAACATATGCATTTTTAAT CATTACACTGAGTAAAGGCATCTC	
		505_F2	51711	AGTGTAATGATTAAAAATGCATATGTTGG GTTAGTTGGTTACATTATAGTGCTG	1933
			5980	GCTGTCAAAGCCAAAGTCATGAGAT	
MBO506	G325A	506_F1	3838	TCTTCTGGCGTGCAGGATTCTTATTAACC	2884
			31478	CTATAATGTAACCAACTAATGCAACATAAC CATTTTTAATCATTACACTGAGT	
		506_F2	51712	GATTAAAAATGGTTATGTTGCATTAGTTGG TTACATTATAGTGCTGTTTAAG	1925
			5980	GCTGTCAAAGCCAAAGTCATGAGAT	
MBO507	G322A + G325A	507_F1	3838	TCTTCTGGCGTGCAGGATTCTTATTAACC	2884
			31479	CTATAATGTAACCAACTAATGCAACATATG CATTTTTAATCATTACACTGAGTAAAGGCA TCTC	
		507_F2	51713	CAGTGTAATGATTAAAAATGCATATGTTGC ATTAGTTGGTTACATTATAGTGCTGTTTAA G	1934
			5980	GCTGTCAAAGCCAAAGTCATGAGAT	
